# An interdisciplinary intervention program to prevent early childhood caries in the Dominican Republic

**DOI:** 10.3389/froh.2023.1176439

**Published:** 2023-09-13

**Authors:** Ninoska Abreu-Placeres, Kim Rud Ekstrand, Luis Eduardo Garrido, Azam Bakhshandeh, Stefania Martignon

**Affiliations:** ^1^ Research Area Cariology and Endodontics, Department of Odontology, Faculty of Health and Medical Sciences, University of Copenhagen, Copenhagen, Denmark; ^2^ Biomaterials and Dentistry Research Center (CIBO-Unibe), Research and Innovation Department, Universidad Iberoamericana, Santo Domingo, Dominican Republic; ^3^ School of Psychology, Pontificia Universidad Católica Madre y Maestra, Santo Domingo, Dominican Republic; ^4^ UNICA—Caries Research Unit, Research Department, Universidad El Bosque, Bogotá, Colombia

**Keywords:** dental caries, pediatrician, prevention, early childhood caries (ECC), oral health

## Abstract

**Objective:**

The principal aim of this randomized clinical trial (RCT) was to test the effectiveness in the prevention of Early Childhood Caries (ECC) through an educational intervention program with the use of a printed guide for pediatricians and parents both designed by pediatric dentists.

**Materials and methods:**

After ethical approval, the first step was to design the educational guides, which were based on the information obtained from a focus group with pediatricians (*n* = 3), phone interviews with mothers to toddlers' (*n* = 7), and the best evidence available about children's oral health. For the RCT, 309 parents with their 10–12 months old children were randomly allocated to either the intervention or the control group. Parents in the intervention group received oral health education from the pediatricians supported by the printed guides. Parents in both groups received an oral health kit with a toothbrush and toothpaste at the first visit as well as at each 6-month follow-up visit. After 18 months the children were evaluated using ICDAS criteria.

**Results:**

At baseline, data were available from 309 children (49.8% girls). The mean age of the children was of 10.8 months (SD = 0.8) and 69.3% had not had their teeth brushed with toothpaste. After 18 months, a total of 28 (22%) children in the intervention group and 44 (24%) in the control group were clinically examined. Regarding the number of tooth surfaces with caries lesions, the children in the intervention group had a mean of 6.50 (SD = 6.58) surfaces, while the children in the control group had a mean of 5.43 (SD = 4.74) surfaces with caries lesions. This difference was not significant (*p* = 0.460).

**Conclusion:**

The RCT showed no effectiveness in caries-progression control. Despite this result, this study managed to identify barriers that do not allow pediatricians from offering parents adequate oral health recommendations. With this learning, it is possible to work on collaborative programs with pediatricians that over time likely will increase dental health by controlling for ECC.

## Introduction

Early Childhood Caries (ECC) has recently been defined as a condition that involves the presence of one or more primary teeth with dental caries lesions -whether cavitated or not, fillings- and missing teeth due to caries, before 6 years of age (1, 2). It is estimated that close to 50% of the world's child population presents ECC according to the WHO criteria, and thus it has been considered as a global health problem (3). Despite lack of robust epidemiological studies in the Caribbean countries, the prevalence of ECC is said to be very high (4–6). For example, in Trinidad and Tobago the mean dmft index in children aged 3–5 in 2014 was 2.83 with a prevalence of dental caries of 50.3% (7).

Umbrella and systematic reviews have highlighted the following risk factors with strong association with development of ECC: caries lesions with compromised dentin, consumption of sugary snacks, inadequate oral hygiene, presence of visible biofilm, enamel defects, among others (8–11). With respect to sugar-rich drinks and their frequent consumption in the bottle, these have been associated with the development of severe ECC caries (12–15). Additionally, night-time feeding practices such as the use of a bottle before going to bed and while the child sleeps have also been related to a high prevalence of dental caries (10). On the other hand, adequate oral hygiene including toothbrushing twice a day with a fluoride toothpaste (≥1,000 ppm F) can be considered as a protective factor for ECC (1, 16).

Family awareness in preventive medicine begins in the physician/pediatrician's office, who provides basic health services from the infant's birth to adolescence (17). Therefore, they are the ideal specialists to begin the oral health education process, instructing on the hygiene routine, promoting good diet habits, and referring to the first visit to the dentist during the first year of age (17–20). Likewise, the pediatrician has the power to include the oral cavity inspection in his physical examination routine, even evaluating the presence of dental caries lesions (18, 19, 21).

Despite having evidence of the impact of oral health on the infant's systemic well-being, barriers have been manifested by the pediatricians to facilitate initial education in preventive dentistry. Thus, insufficient training on the subject has been reported in residency programs (17, 20, 22). This deficiency is detrimental to the health of the child, since studies show that referral to the dentist by the pediatrician increases the chances that this consultation will take place (20, 23). Therefore, establishing such collaborative relationships between pediatricians and dentists at the community level is essential to increase access to dental care for all children and treat early-stage caries lesions (20, 22).

Considering the importance of the prevention of ECC and the role that pediatricians play in it, initiatives have been designed in different countries that seek to integrate the participation of pediatricians and other health professionals in performing activities that allow the prevention and control of dental caries (22, 24, 25). The idea has been to create cooperation between different departments at the hospitals and different health professionals that would work collaboratively to promote oral health in children (22, 24, 25). However, to our current understanding, despite having these initiatives mentioned, no collaborating programs with pediatricians/physicians have been found that are documented in the Caribbean region. Thus, the aim of this randomized clinical trial (RCT) was to test after 18 months the effectiveness in the prevention of Early Childhood Caries (ECC) of an educational intervention program with the use of a printed guide for pediatricians and parents designed by pediatric dentists. A second objective was to implement an oral health program in collaboration with pediatricians in a hospital in the Dominican Republic to educate infants' parents in healthy habits of oral hygiene and diet to prevent the development of dental caries.

## Context

This two-arm randomized clinical trial (RCT), was registered at clinicaltrials.gov (#NCT04101617). The project obtained ethical approval from the hospital and university involved in Santo Domingo, Dominican Republic (DR): (1). Universidad Iberoamericana (CEI# 2017-14), and (2). Hospital General Plaza de la Salud, both in Santo Domingo, Dominican Republic (DR).

## Materials design

**Figure 1:**
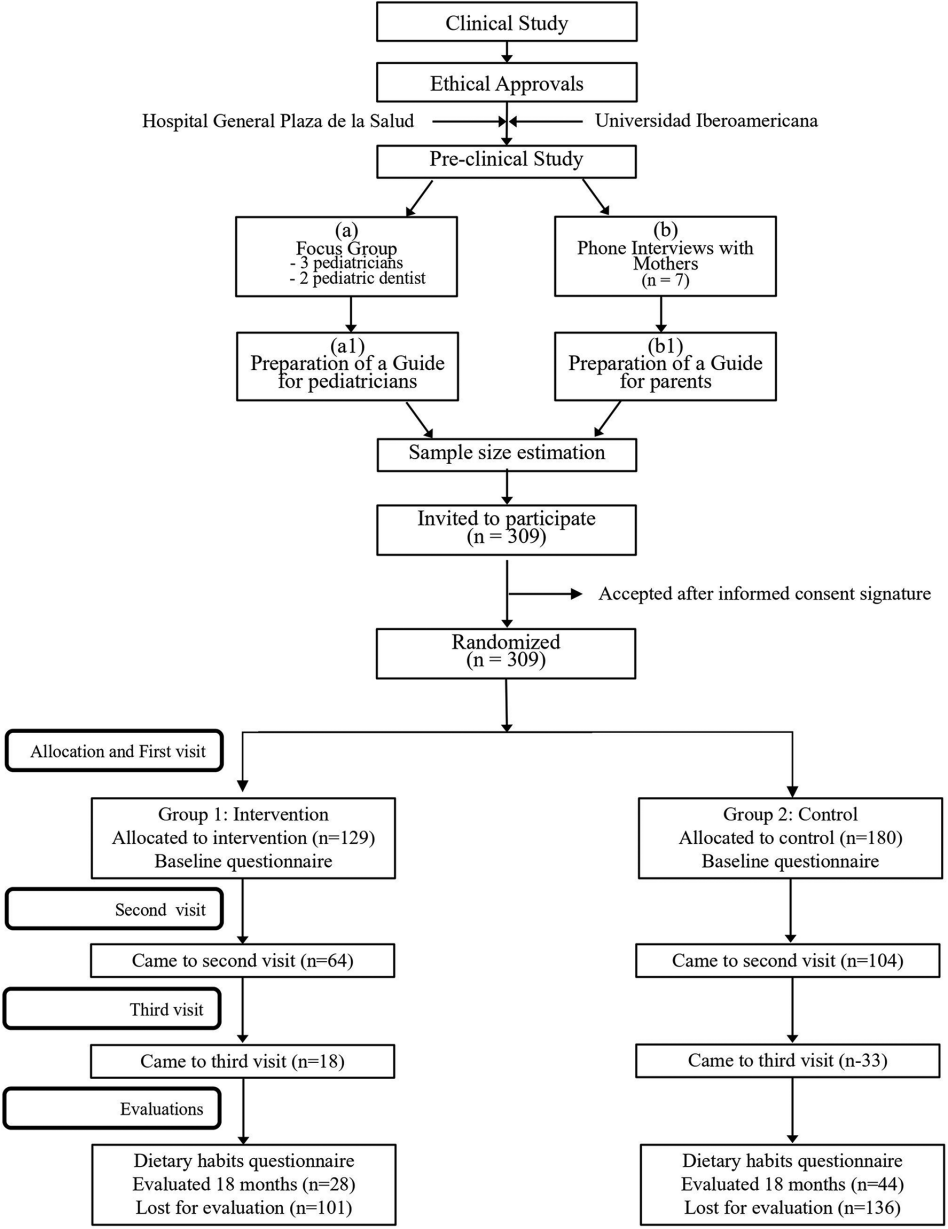
Flowchart of the methodology used in the study.

Before starting the RCT, a pre-clinical study was planned to identify the knowledge, barriers, and behaviors of the pediatricians regarding the provision of oral health education to the parents (Figure 1). Also, to collect relevant information from the mothers to develop an educational program including aid materials (from now on we will call it guide), that the pediatricians were to use during the study to educate the parents. For this purpose, a focus group was carried out with pediatricians and telephone interviews were conducted with mothers.

### Pediatricians' focus groups

A total of 12 practicing pediatricians from different work settings (public, private, and teaching) and with different years since their graduation were invited to participate in a focus group. Of these, three pediatricians agreed to participate and attended the focus group meeting. The objective was to discuss oral health in children and the importance of collaborations between dentists and pediatricians. Semi-structured questions were prepared to obtain the recommendations of the pediatricians regarding how to achieve better oral health for toddlers. Focus group research can be understood through the social constructivist perspective, which highlights the importance of social interactions, group dynamics, and language in shaping individual and collective perspectives (26). This approach emphasizes the social nature of knowledge construction and acknowledges that participants' views are shaped by their social context and interactions with others in the group.

To begin the focus group meeting, the purpose of the activity and the confidentiality of the responses were briefly explained to the participants. After they accepted the terms the focus group started. The complete session was recorded with a rapporteur taking notes and lasting approximately 90 min. The focus group began with an explanation of the relevance of collaboration between both pediatricians and dentists and allowing the pediatricians to an open participation in the discussion. They were questioned about their considerations of oral health and oral hygiene, barriers, and motivations from the pediatricians to perform oral health recommendations to parents. Also, regarding the possibility of giving oral health recommendations to their patient's parents, how they consider it could be more feasible, and if they considered it could be difficult and why (need more time or training). Questions also probed about their availability of time for giving these recommendations and if they considered there was a need of any training related to children's oral health. Finally, we asked about their opinion regarding the development of educational material for guiding them in the process of giving oral health recommendations.

### Mothers' interviews

Using Snowball sampling we identified and interviewed seven mothers from different social environments with at least one child between 10 and 12 months of age. These mothers were contacted through phone calls to set an appointment for semi-structured interviews to be conducted by the principal investigator of this study. The snowball sampling technique is grounded in social network theory, which posits that individuals are embedded within a network of social relationships (27). This technique utilizes this concept by having participants recruit other individuals within their social network to participate in the study. Telephone interviews, according to the interpretive paradigm, enable researchers to view the world through the perceptions and experiences of the participants, gathering rich, in-depth data on their social reality (28).

At the day of the interview, we contacted the mothers and notified them about the interview recording, the confidentiality of the responses and they accepted the terms for the interview. During the interview, the mothers were asked about their family composition, about their children's ages and care, the frequency of the child visits to the pediatrician and their experience with their health care professional. Also, we asked about diet and hygiene daily routine (in general) and more specific questions about the oral health routine (cleaning frequency and moment of the day, and about teeth eruption and primary teeth importance). Additionally, we asked about their pediatrician's oral health recommendations, and barriers and motivation about oral health care and oral hygiene behaviors.

After the focus group meeting and the phone interviews, we began devising two printed guides, one for the pediatricians and the other for the parents. For this process, a literature search was performed to decide the relevant information that was going to be included on the guide. Several guidelines for parents and professionals were consulted to decide what was going to be included. Additionally, we integrated in the guides answers to some of the concerns that the pediatricians and parents gave us during the focus group meeting and phone interviews. The idea was to create a short guide with pictures and very specific information about oral health including oral hygiene and healthy diet. For the pediatrician's guide, pictures showing how dental caries lesions look in their different stages (initial, moderate, and extensive) were included. For the parent's guide, pictures showing the children and parents' positions for the child's toothbrushing at different ages and to show the parents how they must brush their children's teeth.

### Participants and procedure

This study was conducted between February of 2019 and May of 2021. The sample included children between 10 and 12 months old visiting Hospital General Plaza de la Salud (HGPS), in Santo Domingo, for a consultation with a pediatrician. A previously trained research assistant was working with the nurses that were receiving children for consultations. When a child between 10 and 12 months arrived, she invited the parents to participate in the study and explained them the program. Only those children whose parents were literate were considered for the study. If the parents accepted to be enrolled with their children, they had to read and sign the informed consent. After acceptance, a sociodemographic questionnaire (previously piloted with 10 parents) with items about oral health was given to the parents (mother, father, or both that were coming to the hospital with the child). Children with any systemic diseases were not included. Finally, participants were randomly allocated to the intervention or to the control group following a previously prepared list of random numbers in Microsoft Excel.

Participants of the intervention group received a previously prepared printed guide only at the first visit. Then, on the first visit, and every six months or when they had their next visit, participants received oral health and diet education by the previously trained pediatricians, and an oral health kit with a toothbrush and a 1,450 ppm of fluoride toothpaste (1). Participants of the control group received only an oral health kit with a toothbrush and a 1,450 ppm of fluoride toothpaste. No oral examination was performed at baseline because it was assumed that the children did not have visible caries lesions as only few incisors were under eruption (29) at the ages between 10 and 12 months. Additionally, there were no dental examinations performed at the first and second follow-up visits due the difficulty in evaluating children younger than 2 years-old, as well as the substantial increase in costs and logistics associated with it. After eighteen months, the tooth surfaces were evaluated using ICDAS (7 codes criteria) by previously trained pediatric dentists. ICDAS codes greater than 0 were considered as dental caries lesions.

When a new participant joined the trial, the research assistant informed the pediatricians which group they were allocated to. Then, the parents received the adequate recommendations according to their assigned group. Pediatricians also informed the parents about the next health appointment that in accordance with DR regulations is every 6 months for the age group from twelve months to 4 years. Five pediatricians participated in the study guiding the families, and they remained for the duration of the study.

### Sample size calculation

The required sample size was calculated to compare the mean ICDAS caries scores between the intervention and control groups using Welch's independent *t*-test. Welch's *t*-test was chosen because it was assumed that at follow-up the standard deviations of the caries scores could different across groups as a result of the intervention. The sample size calculation was computed based on the following considerations: mean difference in caries experience of 1.25 lesions between the intervention and control group at the 18-months follow-up examination, with an expected standard deviation of 2.5 for the intervention group and 3.5 for the control group. According to Cohen's d measure of effect size for groups with unequal variances (30), this would imply a standardized effect of 0.41, which can be considered as small (31). Power was specified at 80% and the type I error rate at 5% for a two-tailed analysis. The sample size for Welch's independent *t*-test was computed using the R package *sample size* version 0.2–4 (32) with the function *n.ttest*. The sample sizes required were 77 children for the intervention group and 107 for the control group at the end of the study for a total of 184 children. However, adding 40% dropout at the 18-months follow-up, meant that 128 children in the intervention group and 178 in the control group had to be collected, bringing the total enrolment to 306 children.

### Oral evaluations

Appointments were coordinated with the parents for dental prophylaxis and evaluation. When the parents arrived at the appointment, they responded to an interview about oral hygiene and dietary habits of their child. This interview had 4 questions about oral hygiene and 20 questions about dietary habits. The questionnaire employed was prepared by experts in cariology from different countries and belonging to OICAL (Research Observatory for Dental Caries of the Latin American Region), as a project of the Regional Development Program of the International Association for Dental Research (IADR RDP LAR). Furthermore, as the questions were constructed in Spanish, it was only necessary to carry out a cultural adaptation of the questionnaire and a pilot study on 10 mothers to ensure that the parents of the participants understood the questions.

Two blind and ICDAS trained pediatric dentists (examiners) wearing personal protective equipment (PPE) performed the evaluations of the children. One examiner was calibrated in the ICDAS scoring system, and she trained the other dentist. Two sessions were planned for training. For the first session the two examiners discussed the literature and the online material concerning ICDAS criteria. Then, during the second session, both examiners completed the training with a group of 40 extracted teeth for ICDAS criteria detection and discussion. The teeth of the child were cleaned with a rubber cup and prophylactic paste. Then the examiners performed the dental examinations of all the present teeth using a dental mirror, a WHO probe, air syringe, good light, and a saliva ejector. To increase patient cooperation and the reliability of the dental evaluations, the children were sat on top of the parents in the examination chair. Nevertheless, some children (from both study groups) were difficult to evaluate, as is commonly encountered with pediatric patients.

After the evaluation, gel fluoride of 22,500 ppm was painted to the child's teeth. All the parents were informed about their next visit to the pediatric dentist at the hospital according to their child's caries risk. The children who needed dental attention were given an appointment so that the pediatric dentist could see them and solve the necessary dental problems. Oral hygiene recommendations were given to every participant according to their needs. Finally, the printed guide was given to the participants of the control group (since they did not receive it at the time of enrollment) and the oral health kit for the participants of both groups.

### Statistical considerations

Due to the high attrition rate resulting from the COVID-19 pandemic, attrition bias analyses were performed to compare the baseline characteristics of the intervention and control groups remaining at follow-up (33). Group comparisons across continuous variables were performed using Welch's *t*-test (34). With groups of the sizes considered here (*n* ≥ 28), Welch's *t*-test is robust to nonnormality (35–37). For significant *t*-tests, Cohen's *d* measure of effect size was employed, with values of 0.20, 0.50, and 0.80 indicating small, medium, and large effects respectively (31). Regarding group comparisons across categorical variables, they were assessed using the chi-square independence test. For significant chi-square tests, the cells with significant standardized adjusted residuals were identified and Cramer's V index of association reported (38). Cramer's V was interpreted using Cohen's, 1992 guidelines (31), with values of 0.10, 0.30, and 0.50 considered small, moderate, and large, respectively. On the other hand, the weighted kappa statistic with quadratic weights was used to evaluate inter-examiner agreement across the ICDAS ordinal-level scores (39). The following guidelines were used to interpret the kappa values: 0.00–0.20 poor agreement, 0.21–0.40 fair agreement, 0.41–0.60 moderate agreement; 0.61–0.80 substantial agreement; and 0.81 or more excellent agreement (40, 41). All analyses were performed using the IBM software Statistical Package for the Social Sciences (SPSS) version 25. The level of significance for the *p*-values was set at below .05, the conventional cutoff for scientific research.

## Results

### Baseline

A total of 309 children were enrolled in the study. The mean age at baseline was 10.79 months (SD = 0.84), the mean length of breastfeeding was 6.50 months (SD = 3.94). Additionally, the mean age of first tooth eruption was 6.68 months (SD = 1.95), and the mean number of erupted primary teeth was 4.22 (SD = 2.44). The results for the most relevant sociodemographic variables are as follows: Sex: 50.2% were boys. Educational level of the parents: 46.6% of the mothers and 34.3% of the fathers had university degrees, and all had finished primary school. Monthly income level (Dominican pesos): 41.8% earned 30,000 or less a month, 24.5% between 30,001 and 50,000, and 33.7% more than 50,000. Toothbrushing: of the 285 children with visible teeth at the first visit, 68.8% never used toothbrush, 4.2% less than once per day, 18.2% once a day, and 8.8% more than once a day. Uses of toothpaste: 75.4% of the parents did not brush their children's teeth with toothpaste. Parents “own caries experienced: 23.8% of the mothers and 29.2% of the fathers had never experienced dental caries”. Nationality: Nearly all mothers and fathers were from the Dominican Republic (>98%). Finally, nearly all children (99.0%) had health insurance.

### Final visit and clinical examination

A total of 28 (21.7%) children in the intervention group and 44 (24.4%) in the control group were clinically examined 18 months after the enrolment in the study. With sample sizes of 28 and 44 for the two groups, 5% type I error, 80% power, and for a two-tailed analysis, the G*Power3 software indicated that a standardized mean difference of *d* = 0.69 or greater could be detected. According to Cohen, 1992 (31), this effect size could be categorized as medium-sized. Attrition analyses showed that the children that continued in the study (*n* = 72) did not differ from the children that dropped out (*n* = 237) across any of the variables evaluated at baseline. Welch's *t*-test was employed to compare the two groups across the following continuous variables: age of the children (*p* = .094), time breast-feeding (*p* = .357), number of erupted teeth (*p* = .910), and age of first tooth eruption (*p* = .918). Similarly, chi-square independent tests were performed to compare the groups across the following categorical variables: study group (*p* = .574), sex (*p* = .612), mother educational level (*p* = .647), father educational level (*p* = .471), illness during pregnancy (*p* = .577), medication during pregnancy (*p* = .937), monthly house income (*p* = .532), toothbrushing frequency (*p* = .894), use of toothpaste (*p* = .679), mother dental caries experience (*p* = .665), and father dental caries experience (*p* = .903).

At the follow-up examination, the children in the intervention group had approximately equal number of erupted teeth (*M* = 19.07, SD = 1.56) compared to the children in the control group [*M* = 19.05, SD = 1.51, *t* (56.16) = 0.07, *p* = .945]. Regarding the dental caries assessment, the weighted kappa statistic for the inter-examiner agreement on the ICDAS scores (0–6 scale) across 136 surfaces was 0.84 (*p* < .001), indicating excellent agreement. In terms of the number of teeth with caries lesions, the difference between the children in the intervention group (*M* = 5.04, SD = 2.99) and those in the control group (*M* = 4.43, SD = 2.85) was not statistically significant [*t* (55.59) = 0.85, *p* = .398]. Similarly, the number of tooth surfaces with caries lesions for the children in the intervention group (*M* = 6.50, SD = 6.58) was not significantly different from those of the children in the control group [*M* = 5.43, SD = 4.74, *t* (44.69) = 0.75, *p* = .460].

In terms of the severity of the caries lesions across surfaces, the results indicated that the mean scores on the ICDAS 0–6 scale of the intervention group (*M* = 0.15, SD = 0.28) and the control group (*M* = 0.10, SD = 0.12) were not significantly different [*t* (33.38) = 0.85, *p* = .403]. Another severity criterion examined for the caries lesions was the maximum ICDAS score for each child. According to this criterion, the children in the intervention group (*M* = 1.93, SD = 0.94) did not have a significantly different caries experience compared to those in the control group [*M* = 1.75, SD = 1.22, *t* (67.38) = 0.70, *p* = .488].

**Figure 2:**
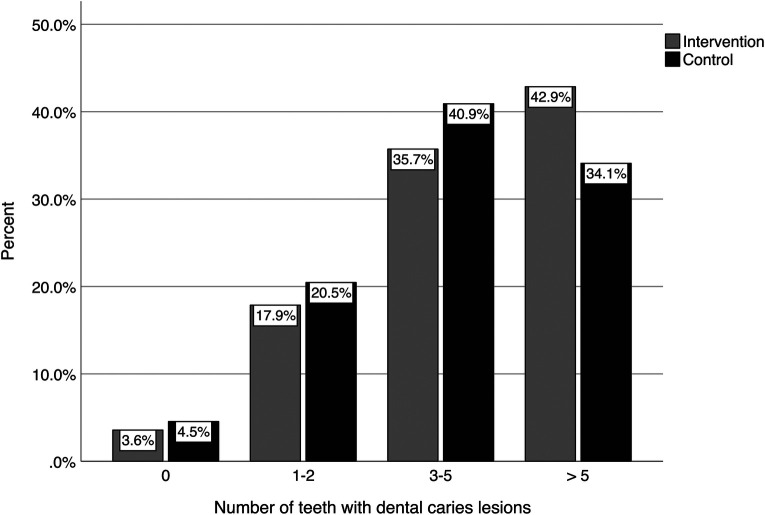
Number of teeth per child with dental caries lesions at follow-up.

**Figure 3:**
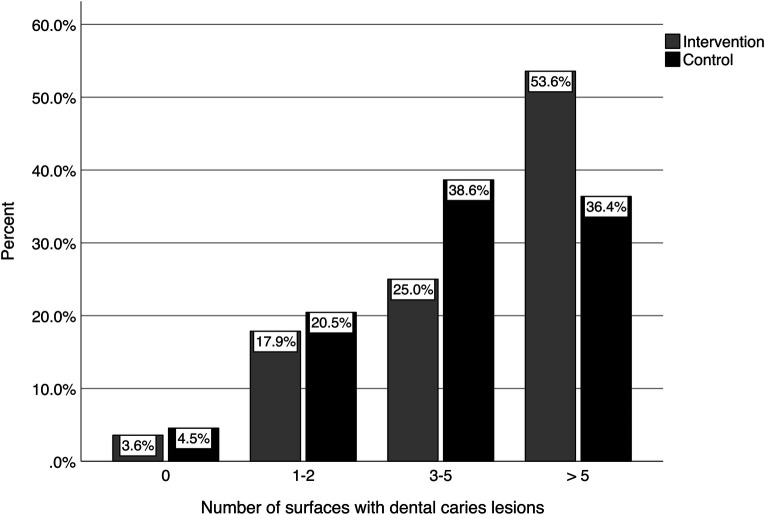
Number of tooth surfaces per child with dental caries lesions at follow-up.

The distributions of the number of teeth with caries lesions for the intervention and control groups are shown in Figure 2. As can be seen in the figure, less than 5% of the children of both groups had no caries lesions at follow-up, while more than 70% had three or more teeth with caries lesions. Figure 3 shows the distribution of the number of tooth surfaces with caries lesions. These results were similar to those at the tooth level, with more than 70% of the children having three or more surfaces with caries lesions.

**Figure 4:**
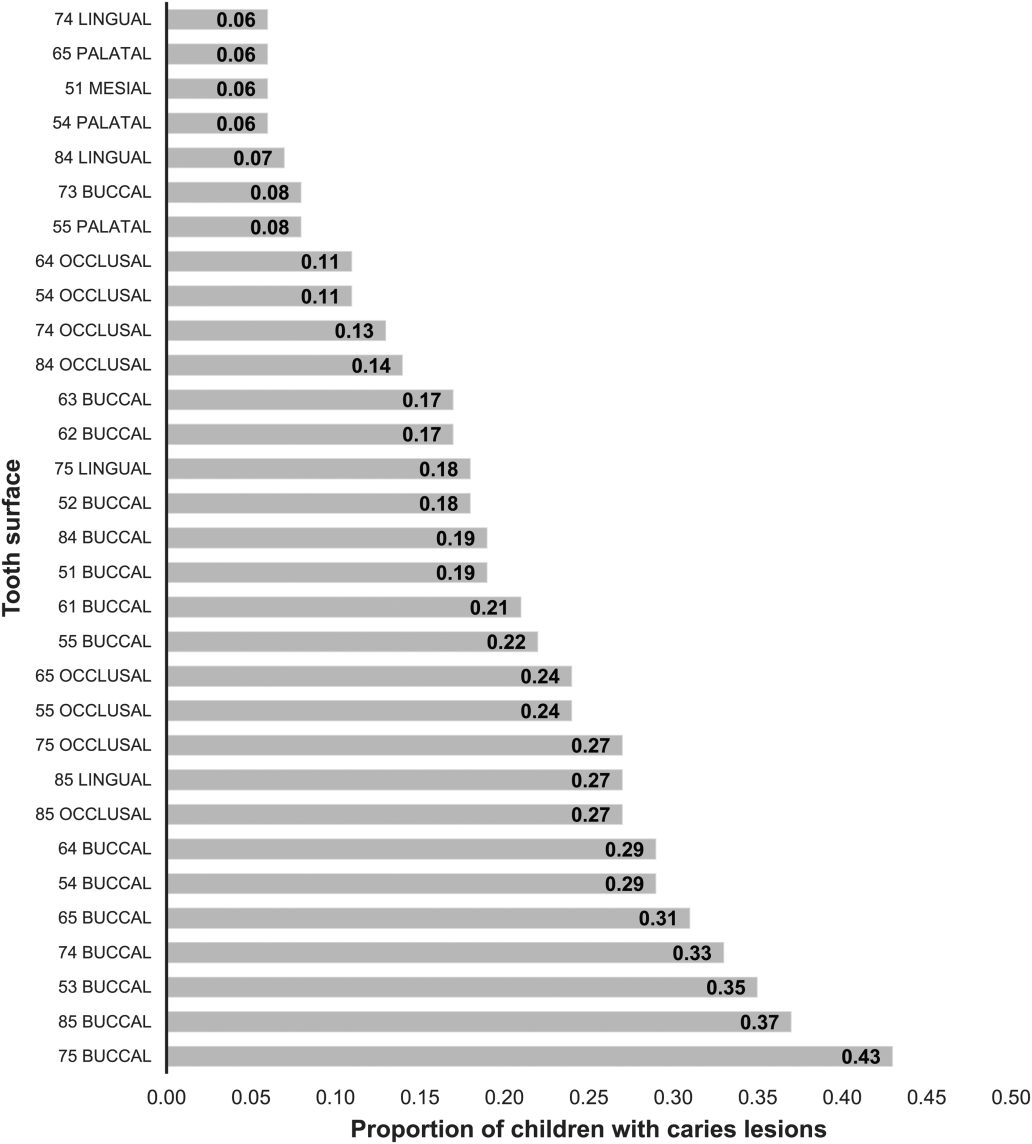
Proportion of children with caries lesions across the tooth surfaces. Tooth surfaces with less than 5% caries lesion rates are not included in the graph. FDI World Dental Federation tooth numbering system for primary teeth was used.

The proportion of children with caries lesions across the tooth surfaces is shown in Figure 4. Of the 88 tooth surfaces in the primary dentition, 39 (44.3%) did not exhibit caries lesions, while 18 (20.5%) had caries lesion rates between 1% and 4%. The surface with the highest caries lesion rate was the buccal surface and belonged to the tooth #75. This surface had caries lesions in 43% of the children examined at follow-up.

The toothbrushing and dietary habits of the intervention and control groups were compared using chi-square independent tests. These analyses revealed that children in the control group drank sugary beverages in a cup more often (72.7% drank them more than once a day) than the children in the intervention group (32.1% drank them more than once a day; $\;\chi^{2}\;[2]\;=\;14.67$, *p* < .001). For the rest of the variables examined there was no significant association with group membership (.128 ≤ *p *≤ .975). Across the complete follow-up sample, 68.1% of the children had their teeth brushed two or more times a day, 97.1% always used fluoridated toothpaste, and only 40.3% had their teeth brushed always or most of the time before going to bed.

## Discussion

ECC is a major oral health problem around the world, including the DR (6, 42). Dental caries and especially ECC have a substantial impact on the quality of life of children and their families (6, 43). To prevent ECC, a multi-disciplinary team approach that emphasizes interprofessional collaboration between dental professionals, pediatricians, and other healthcare providers is crucial. Each team member brings unique skills and knowledge, enabling them to provide effective care. In particular, pediatricians play a pivotal role in promoting good oral health practices among children and their caregivers, making them an essential part of the team. In the DR there is no prevention program in the dental health system, and dental care is not free of charge. This is in contrast, for example, with the Public Pediatric Dental Healthcare System in the Scandinavian countries, which has an obligation to contact the parents shortly after the child is born. Such efforts establish good cooperation between parents and the dentist as early as possible for the benefit of the child. Several attempts have been undertaken to implement similar cooperation with pediatricians around the world (22, 24, 25) but to the best of our knowledge the effects have not been evaluated in any RCTs.

In this study, two educational guides were designed, one for pediatricians and the other for the parents of toddlers. These guides were devised considering the relevant information on oral health in childhood, and the responses obtained in the focus group with the pediatricians and the mothers' interviews. The recommendations of the pediatricians were included in the guides. Similarly, pictures of the toothbrushes and the amount of toothpaste to be placed were included as well as the recommended toothbrushing technique depending on the age of the child. It was also sought to guide parents with examples about the position in which they can place the child when toothbrushing and the recommended toothbrushing technique according to the number of teeth that their child had. Nevertheless, to mobilize change in children's oral health care, better communication between pediatricians and family caregivers is important. Pediatricians are the first point of contact for parents and caregivers of young children, so they can clarify doubts and provide them with clear and accurate information about dental caries prevention. Pediatricians can also recommend regular dental checkups for children to detect any early signs of dental caries (18, 21).

Of the participants who completed the study, the percentage of toddlers who received toothbrushing increased from 34.8% at baseline to 95.8%, so that only 4.2% of the participants had parents not brushing the child's teeth at the follow-up. Similarly, regarding the use of fluoride toothpaste, there was an increase from 20.8% to 97.1%, with only 2.9% not using fluoride toothpaste at the follow-up examination. These results show that there was an increase in the frequency of toothbrushing and the use of fluoride toothpaste, an action that could have been influenced by the toothbrush and toothpaste that the parents received from an early age as part of the program.

Regarding the children's diet at follow-up, 83.1% of the children were still drinking milk from the bottle, and the children consumed fruit juices with added sugar. In this case, it is relevant to mention that an increase in ECC has been reported in children who are bottle-fed at 12 months of age or more (15). For the participants in this study, the mean age at the time of the dietary habit's measurement was 30.8 months, which may also explain the reported experience of dental caries. It has been reported that the consumption of beverages and foods with added free sugars plays an important role in the development of dental caries lesions and ECC (1, 12, 13). This could explain why some participants, despite regular toothbrushing, had active caries lesions in some of their teeth. Also, this makes us reconsider the way the message should be carried to parents to avoid the consumption of free sugars since this leads to an increased risk of dental caries in children. In this regard, Feldens et al. 2021 suggest that strategies need to be developed to promote healthy eating habits during the first year of life (44).

The results of this RCT showed no significant differences between the intervention and control groups in terms of the severity of dental caries lesions, both at the tooth and surface level. An aspect to distinguish is that all the lesions assessed by the examiners were active lesions. This is because they were mostly found in biofilm stagnation areas (45). Despite these results, it is relevant to mention that this type of program helps to educate parents in healthy oral hygiene and eating habits and also it can support pediatricians in the training of the parents to achieve long-term prevention.

An important challenge in this study was to achieve the needed motivation of the pediatricians to offer oral health recommendations to the participants and their parents. It should be noted that one of the payment mechanisms in many hospitals is fee-for-service, including the one where this study was carried out. That is, the professional receives a payment for each patient he/she attends, which implies that they have a minimum of patients to see per week and the need to consult more patients in less time. This leads us to think about how we can motivate pediatricians given the current work and payment system to ensure that they consider oral health in their evaluations and recommendations.

Another barrier that can be mentioned is that the dental trainings that pediatricians receive in their pediatric residencies are limited. This situation has already been well documented in other countries (3, 46, 47). Also, it was a topic of discussion in the focus group of this study, since the participating pediatricians were teachers of a pediatric postgraduate program, and they require that their residents follow the same protocol as them. This means that the pediatrician could not have the necessary capability to give oral health recommendations and teach the parents of their patients the necessary hygiene habits (47). Considering all the points raised above, it becomes clear that these barriers that pediatricians face in their day-to-day life must be identified. In addition, it is necessary to assess how they influence their behaviors of giving healthy habits recommendations, in order to design effective interventions that help achieve a behavior change. To address this issue, we recommend future research using the behavior change wheel and its COM-B model (48). This would allow to inquire about the capacity, opportunity, and motivation that pediatricians have to offer oral health recommendations to their patients and could help identify which are the components that influence behavior and focus the strategies for change in those.

Furthermore, this program allowed the identification of some barriers and facilitators that can be the basis for the development of future strategies for the prevention of ECC. Additionally, from the experience gained in the implementation of this program and the faced difficulties, we are currently working in DR to introduce an oral health program for children in the pediatric residences of the hospitals. This project is being carried out with the collaboration of all the stakeholders, including the Ministry of Health and the Ministry of Higher Education of the country. Therefore, it would be possible for residents to receive the necessary training in oral health and to include it in their patient care protocol.

This study had some limitations that must be underlined. The most important was the size of the sample at follow-up. Although the sample was sufficiently powered to detect small mean differences in caries experience, and even though a large dropout rate of 40% was incorporated into the sample size calculations, the COVID-19 pandemic constituted a major obstacle for this study. Nevertheless, additional power analysis computations showed that with the actual sample sizes collected, medium-sized differences in caries experience could still be detected, supporting the utility of the conducted analyses, albeit at a reduced power level. Importantly, extensive attrition analyses were performed, and these showed that the group that did not return for the follow-up evaluation was approximately equal to the group that remained in the study, evidence suggesting reduced potential for bias. This further supports the validity of the findings of the study.

## Conclusions

This study provided a formative and insightful experience regarding collaborative programs between pediatricians and dentists. With it, it was possible to identify some barriers, facilitators and aspects to ensure that pediatricians educate the parents of their patients in healthy oral hygiene and eating habits. These findings can help the decision-making of the different stakeholders and help design effective strategies to achieve a behavior change in pediatricians and therefore achieve an improvement in the oral health of children and their families. A well-planned program in conjunction with pediatricians and dentists where there is sufficient motivation and training could have the potential to help reduce the probability of developing ECC.

## Data Availability

The raw data supporting the conclusions of this article will be made available by the authors, without undue reservation.
